# Genome-wide identification and expression analysis of histone deacetylase and histone acetyltransferase genes in response to drought in poplars

**DOI:** 10.1186/s12864-024-10570-1

**Published:** 2024-07-02

**Authors:** Huanhuan Li, Yao Chen, Yujie Dai, Le Yang, Sheng Zhang

**Affiliations:** Key Laboratory of Bio-Resource and Eco-Environment of Ministry of Education, College of Life Sciences, Sichuan University, Chengdu, 610065 China

**Keywords:** Histone deacetylase, Histone acetyltransferase, Drought, Hormones, *Populus*

## Abstract

**Background:**

Histone deacetylases (HDACs) and histone acetyltransferases (HATs) are involved in plant growth and development as well as in response to environmental changes, by dynamically regulating gene acetylation levels. Although there have been numerous reports on the identification and function of HDAC and HAT in herbaceous plants, there are fewer report related genes in woody plants under drought stress.

**Results:**

In this study, we performed a genome-wide analysis of the *HDAC* and *HAT* families in *Populus trichocarpa*, including phylogenetic analysis, gene structure, conserved domains, and expression analysis. A total of 16 *PtrHDACs* and 12 *PtrHATs* were identified in *P. trichocarpa* genome. Analysis of *cis*-elements in the promoters of *PtrHDACs* and *PtrHATs* revealed that both gene families could respond to a variety of environmental signals, including hormones and drought. Furthermore, real time quantitative PCR indicated that *PtrHDA906* and *PtrHAG3* were significantly responsive to drought. *PtrHDA906, PtrHAC1, PtrHAC3, PtrHAG2, PtrHAG6* and *PtrHAF1* consistently responded to abscisic acid, methyl jasmonate and salicylic acid under drought conditions.

**Conclusions:**

Our study demonstrates that *PtrHDACs* and *PtrHATs* may respond to drought through hormone signaling pathways, which helps to reveal the hub of acetylation modification in hormone regulation of abiotic stress.

**Supplementary Information:**

The online version contains supplementary material available at 10.1186/s12864-024-10570-1.

## Background

It has been demonstrated that plants have evolved epigenetic mechanisms to maintain stable growth and development under different environmental conditions [1]. Histone modification is one of the important epigenetic regulations of higher plants to adapt to environmental changes [2]. The most prevalent histone modification is histone acetylation, which is catalyzed by both histone deacetylases (HDACs) and histone acetyltransferases (HATs) [3]. HATs promote histone acetylation, which neutralizes the positive charge of histone tails and reduces their affinity for negatively charged DNA. This increases promoting chromatin accessibility to transcriptional regulators and facilitates gene expression [4, 5]. Conversely, the removal of acetyl groups by histone deacetylases can result in more compact chromatin, thereby repressing gene expression [6, 7].

HDACs can be divided into three subfamilies in plants, including the reduced potassium dependency 3/histone deacetylase 1 (RPD3/HDA1), histone deacetylase 2 (HD2), and silent information regulator 2 (SIR2) [8, 9]. They play important roles in ovule and seed developments, and regulate the equilibrium between plant growth and stress response by regulating rRNA processing [10, 11]. In parallel, plant HATs have been classified into four groups, including GCN5-related N-terminal acetyltransferase (GNAT), CREB-binding protein (CBP), MOZ, Ybf2/Sas3, Sas2 and Tip60 superfamily (MYST), and TATA-binding protein-associated factor (TAFII250) [7]. These proteins are involved in the modification of chromatin structure, the regulation of gene expression, and DNA repair [12, 13].

HDACs and HATs play important roles in the response to drought stress. For example, in *Arabidopsis thaliana*, drought stress resulted in a significant enrichment of H3K9ac at the promoters of genes (*RD29A, RD29B*, and *RD20*) involved in drought response, thereby activating their expression [14]. The mutation of *PtrGCN5* would result in an increased sensitivity of *Populus trichocarpa* to drought [15]. In *Oryza sativa*, OsHDA704 was found to bind to DST and ABIL2, resulting in their transcriptional repression through deacetylation. This process negatively regulates stomatal opening and density, thereby enhancing drought tolerance [16]. Concurrently, OsHDA704 reduced the acetylation levels of *OsABI5* and *OsDSS1* by recruiting OsWR2, thereby enhancing drought tolerance [17]. Furthermore, plants utilize endogenous hormone synthesis and hormone signaling pathways to enhance their drought tolerance. AtHDA6 functions as a drought regulator promoting the biosynthesis of acetic acid, stimulating the initiation of the JA signaling pathway, and enhancing the drought resistance in *A. thaliana* [18]. AtHDA15 enhances plant drought tolerance by inhibiting negative regulators of abscisic acid (ABA) signaling, following recruitment by AtMYB96 [19]. In addition, the overexpression of the *84 K* (*Populus alba × Populus glandulosa*) gene *HDA909* has been demonstrated to enhance plant drought tolerance by upregulating the expression of the stress-responsive genes, including *HK1* and *ZAT12*, and the accumulation of ABA [20]. The previous studies have demonstrated that histone acetyltransferases and deacetylases are crucial for plant responses to drought.

Current studies on the identification of the HDAC and HAT families have focused on herbaceous plants. However, relatively few studies have been conducted on woody plants. In this study, the genome-wide analysis of *HDACs* and *HATs* was performed in *P. trichocarpa*. A comprehensive investigation was conducted to ascertain chromosomal localization, gene structure, conserved motifs, phylogenetic relationships, and putative *cis*-elements in the promoters of the *PtrHDACs* and *PtrHATs*. Furthermore, the expression patterns of *PtrHDACs* and *PtrHATs* in response to different hormone treatments under drought conditions were analyzed. The objective of this study was to elucidate the potential roles of *PtrHDACs* and *PtrHATs* in hormone-regulated drought responses in poplar.

## Results

### Identification of HADC and HAT family members in *P. trichocarpa*

**Fig. 1:**
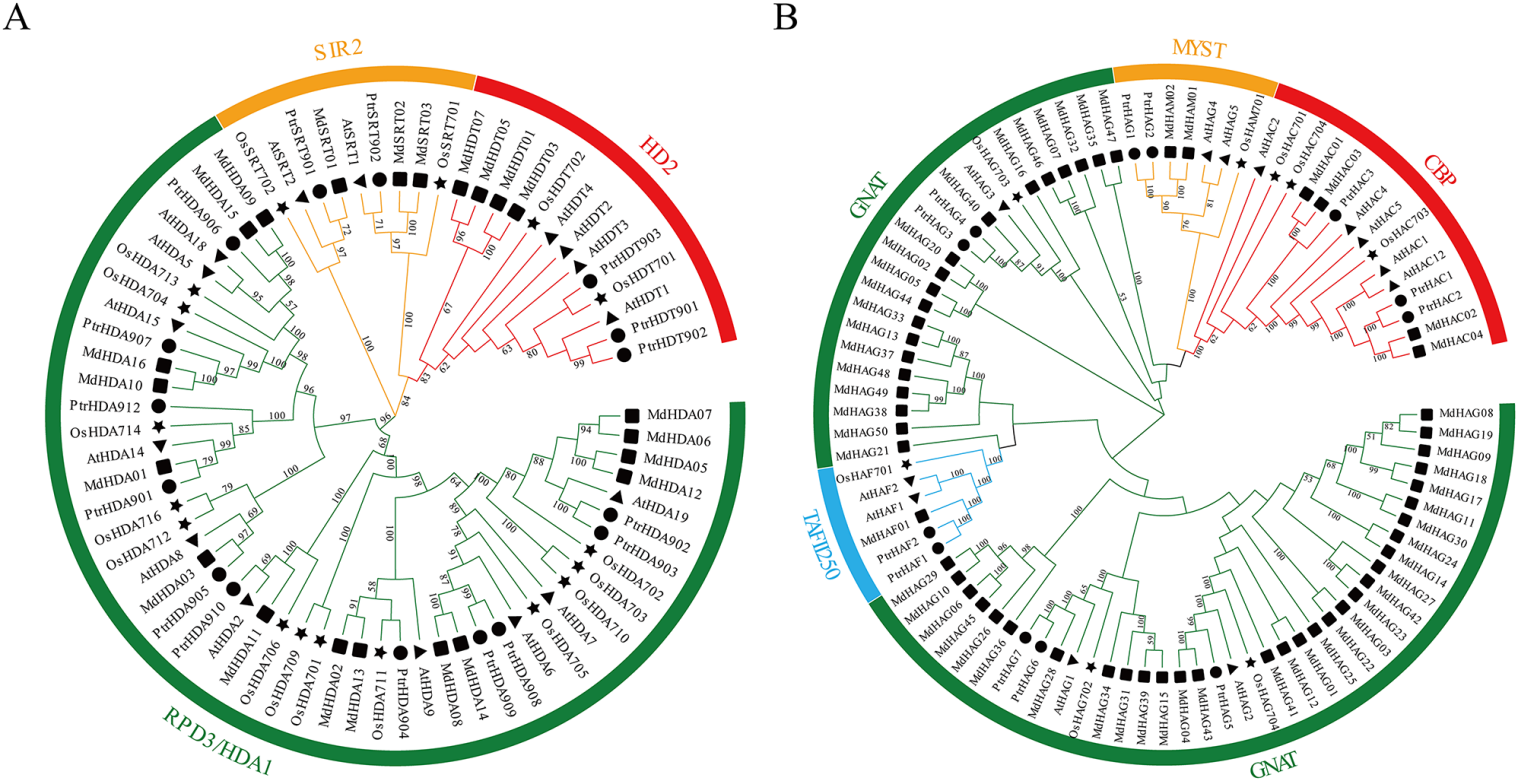
Evolutionary and phylogenetic analysis of the *HDAC* (**A**) and *HAT* (**B**) genes family in *A. thaliana*, *O. sativa, M. domestica* and *P. trichocarpa*. Different shapes represent different species. The black triangle is *A. thaliana*. The black star is *O. sativa*. The black rect is *M. domestica.* The black circle is *P. trichocarpa*. The unrooted phylogenetic tree is constructed with MEGA 11 using the neighbor-joining method and the bootstrap analysis is performed with 500 replicates. The colors of the outer ring and branches indicate different group

A total of 16 *PtrHDACs* and 12 *PtrHATs* were identified in *P. trichocarpa* (Fig. 1; Table 1). Phylogenetic analyses were constructed using the protein sequences of the HDAC and HAT families on *A. thaliana*, *O. sativa*, *Malus domestica*, and *P. trichocarpa*, respectively, to investigate the evolutionary relationship and classification (Fig. 1). The results showed that the 16 members of HDAC proteins in *P. trichocarpa* could be classified into three subfamilies, including 11 members of the RPD3/HDA1 subfamily, 3 members of the HD2 subfamily, and 2 members of the SIR2 subfamily. The 12 HAT members were categorized into four subfamilies, with 5 members of the GNAT subfamily, 2 members of the MYST subfamily, 3 members of the CBP subfamily, and 2 members of the TAFII250 subfamily.

**Table 1:** Overview of the histone deacetylases and acetyltransferase genes identified in *P. trichocarpa*

Subfamily	Gene name	Gene ID	Protein length	Molecular weight (kD)	Isoelectric point	Localization
*HDACs family*						
RPD3/HDA1	*PtrHDA901*	Potri.005G064200	414	45.08	5.77	chloroplast
	*PtrHDA902*	Potri.009G170700	499	56.18	5.06	nucleus
	*PtrHDA903*	Potri.004G209800	501	56.13	5.26	nucleus
	*PtrHDA904*	Potri.001G460000	429	48.85	5.06	chloroplast
	*PtrHDA905*	Potri.009G020500	379	41.17	5.92	cytoplasm
	*PtrHDA906*	Potri.004G092900	655	73.27	5.35	cytoskeleton
	*PtrHDA907*	Potri.012G060400	594	64.92	6.29	nucleus
	*PtrHDA908*	Potri.015G082500	467	52.33	5.36	nucleus
	*PtrHDA909*	Potri.012G083800	440	49.51	5.28	nucleus
	*PtrHDA910*	Potri.006G230300	391	43.41	8.07	chloroplast
	*PtrHDA912*	Potri.007G104900	144	15.63	4.65	cytoplasm
HD2	*PtrHDT901*	Potri.009G149400	275	29.65	4.71	nucleus
	*PtrHDT902*	Potri.004G188800	279	30.16	4.70	nucleus
	*PtrHDT903*	Potri.006G116500	305	33.35	4.61	nucleus
SIR2	*PtrSRT901*	Potri.005G068400	387	42.85	9.41	chloroplast
	*PtrSRT902*	Potri.001G368100	464	51.67	8.94	peroxisome
*HATs family*						
MYST	*PtrHAG1*	Potri.005G171700	448	52.08	6.56	nucleus
	*PtrHAG2*	Potri.002G089500	445	51.83	7.20	nucleus
GNAT	*PtrHAG3*	Potri.012G092800	563	63.34	8.76	cytoplasm
	*PtrHAG4*	Potri.015G090500	563	63.24	8.83	cytoplasm
	*PtrHAG5*	Potri.013G068200	463	52.13	5.67	nucleus
	*PtrHAG6*	Potri.005G217400	600	66.87	6.22	nucleus
	*PtrHAG7*	Potri.002G045900	556	61.89	6.11	nucleus
CBP	*PtrHAC1*	Potri.014G000500	1736	195.87	8.50	nucleus
	*PtrHAC2*	Potri.007G003400	1421	161.85	8.04	nucleus
	*PtrHAC3*	Potri.004G104201	1463	165.87	6.92	nucleus
TAFII250	*PtrHAF1*	Potri.017G047600	1902	214.88	6.12	nucleus
	*PtrHAF2*	Potri.007G113300	1895	214.75	6.16	nucleus

A physicochemical analysis of the identified genes was conducted using the ProtParam website. The proteins belonging to the PtrHDAC family ranged in length from 144 to 655 amino acids and in molecular weight from 15.62 to 73.27 kD. The theoretical isoelectric point (pI) of the PtrHDACs exhibited a range of values, from 4.61 to 9.41. The proteins within the PtrHAT family exhibited a range of lengths, from 445 to 1902 amino acids, and molecular weights, from 51.83 to 214.88 kD. The theoretical isoelectric point (pI) of the PtrHATs ranged from 5.67 to 8.83. With regard to subcellular localization, the majority of PtrHDACs were identified in the nucleus and in the chloroplast, as well as in the cytoplasm and nucleus, whereas PtrHATs were found to be located in the nucleus and cytoplasm.

### Phylogenetic tree analysis, motif and gene structure of PtrHDACs and PtrHATs

**Fig. 2:**
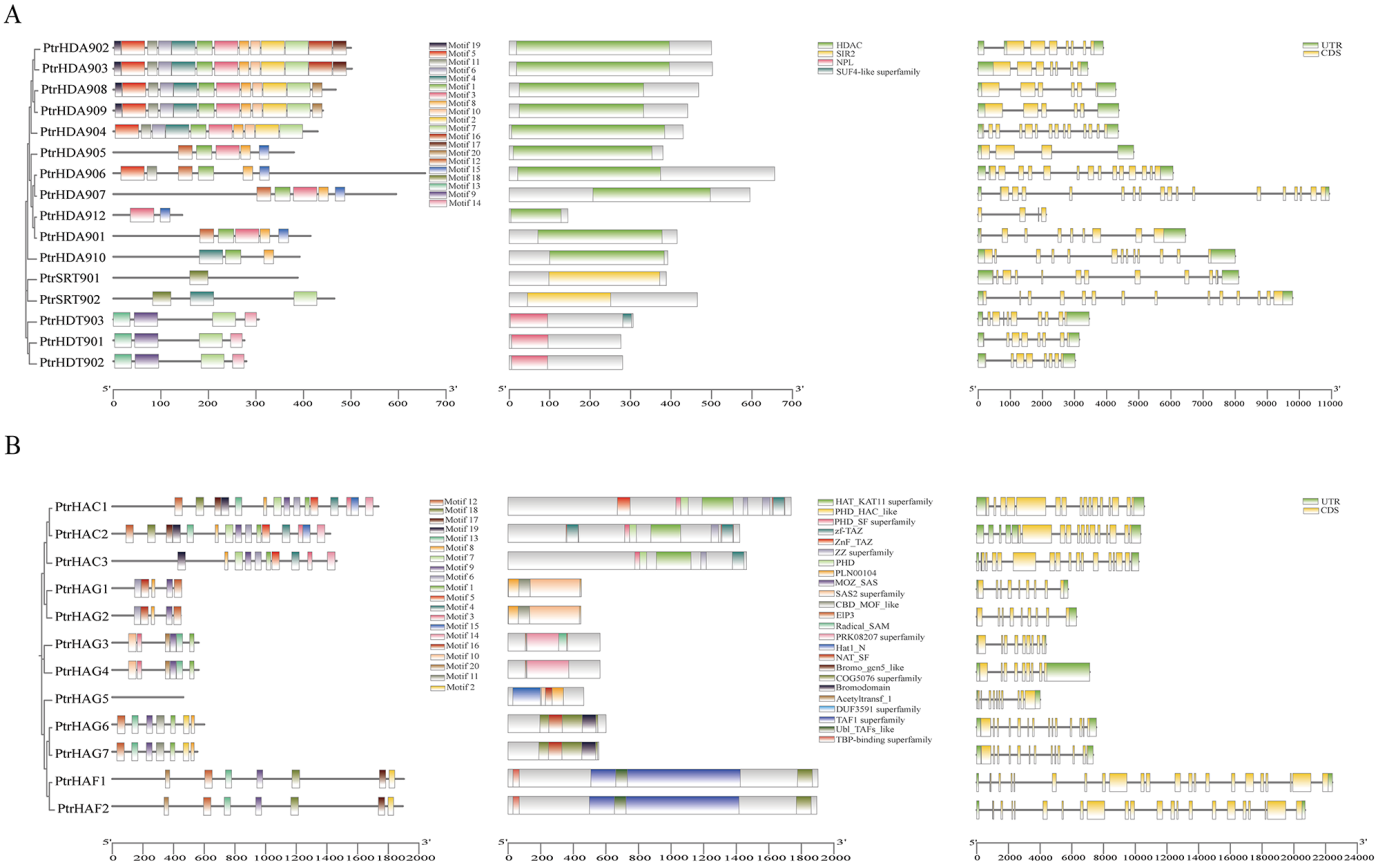
Motifs and gene structure of PtrHDACs (**A**) and PtrHATs (**B**). From left to right, the phylogenetic tree, motif distribution, domain organization and gene structure of PtrHDACs and PtrHATs are shown

The phylogenetic tree analysis of the PtrHDACs or PtrHATs family showed the same results as in Fig. 1. The MEME program was employed to predict the conserved motifs of PtrHDACs, and a total of twenty conserved motifs were identified (Fig. 2A). A structural domain analysis revealed that different HDAC subfamilies contained specific conserved domains (Fig. 2A). The results showed that the RPD3/HDA1 subfamily contained Motif1 and Motif8, and that members of the RPD3/HDA1 subfamily contained HDAC structural domains. Furthermore, Motif9, Motif13 and Motif14 were observed to be exclusive to the HD2 subfamily, with Motif9 and Motif13 playing a role in the formation of the N-terminal NPL domain. In addition, the SIR2 subfamily exhibited a specific Motif 18, while PtrSRT902 further displayed two motifs (Motif 4 and Motif 7) in comparison to PtrSRT901. Both members of the SIR2 subfamily exhibited conserved structural domains characteristic of the SIR2 protein family.

Similarly, 20 conserved motifs were identified in the PtrHAT family (Fig. 2B). In the GNAT subfamily, except for PtrHAG5, all members contained Motif13, Motif1, which may contain the Hat1_N or acetyltransferase or radical SAM domain. Motif16 was only found in the MYST subfamily. Members of this subfamily contained both the MOZ_SAS and SAS superfamily domains. Members of the CBP subfamily exhibited the characteristic Motif 4, Motif 5, Motif 7, Motif 14, Motif 19, as well as the HAT-KAT11, PHD, and ZZ domains. All members of the TAFII250 subfamily exhibited the presence of Motif2, Motif12, Motif17, Motif18, and Motif20. Additionally, this subfamily contained the DUF3591 and Bromodomain. With the exception of PtrHAG5, all members of the PtrHATs family exhibited the presence of Motif9. Furthermore, the intron–exon organization of these *PtrHATs* and *PtrHDACs* was analyzed. The number of conserved coding regions ranged from 9 to 21 in *PtrHATs* and from 3 to 17 in *PtrHDACs*.

### Chromosomal location and duplication of *PtrHDACs* and *PtrHATs*

**Fig. 3:**
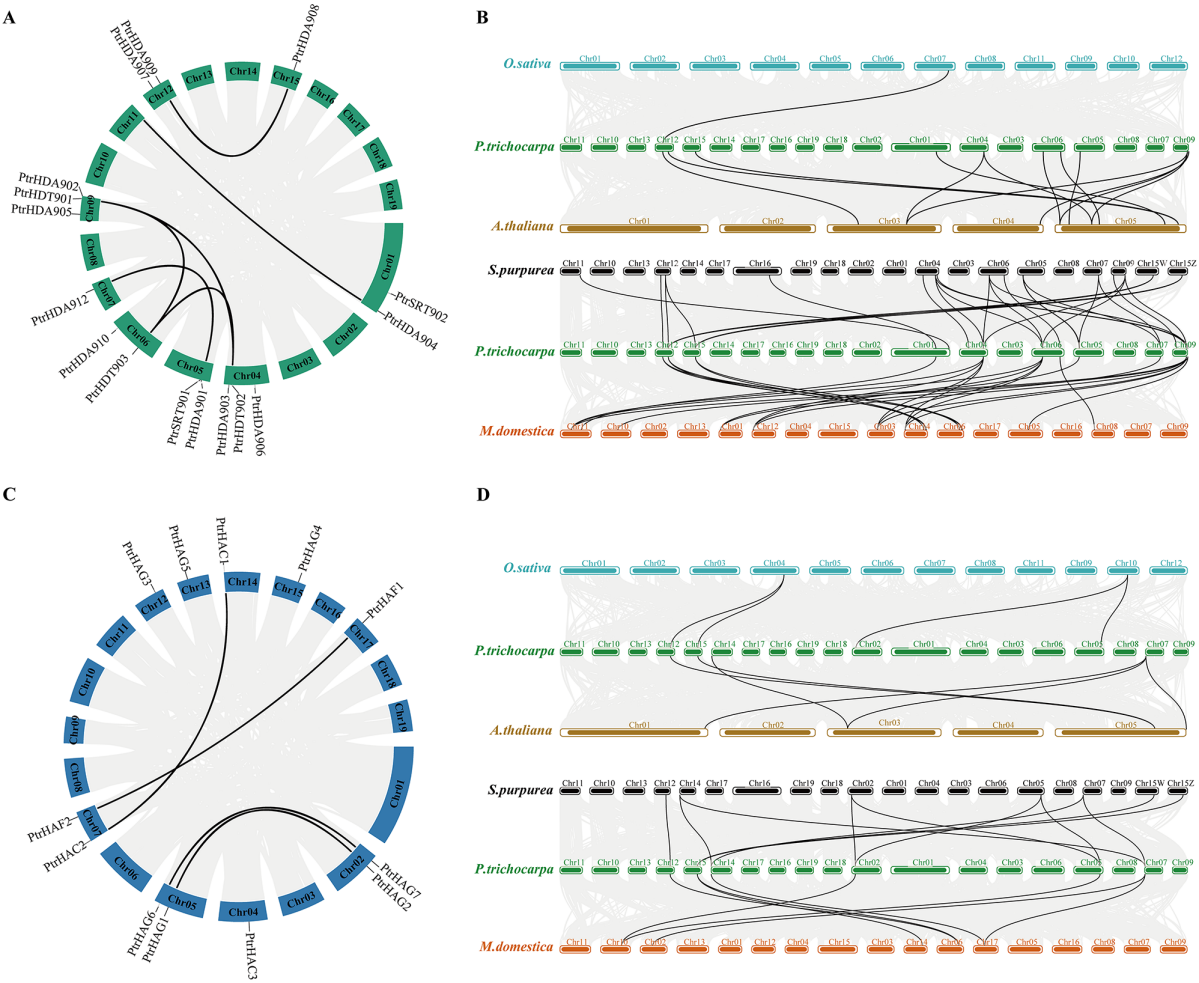
Intraspecific and interspecific collinearity analysis of *HDACs* and *HATs*. Intraspecific collinearity of *PtrHDACs* (**A**). Interspecific collinearity analysis (**B**) of *PtrHDACs* against *A. thaliana* and *O. sativa*, or against *S. purpurea* and *M. domestica*. Intraspecific collinearity of *PtrHATs* (**C**). Interspecific collinearity analysis (**D**) of *PtrHATs* against *A. thaliana* and *O. sativa*, or against *S. purpurea* and *M. domestica*

The genome annotation information was used to create a chromosome map of *PtrHDACs* and *PtrHATs* was drawn, with all 28 members displaying specific locations (Fig. 3). The chromosomal distribution of *PtrHDACs* was found to be irregular, with the genes present on chromosomes 1, 4, 5, 6, 7, 9, 12 and 15 (Fig. 3A). Specifically, chromosomes 1, 5, 6, and 12 exhibited the presence of two *PtrHDACs*, while chromosome 4 and 9 exhibited the presence of three *PtrHDACs*. *PtrHDA912* and *PtrHDA908* were distributed on chromosomes 7 and 15. Similarly, the 12 *PtrHAT* members were predominantly distributed on chromosomes 2, 4, 5, 7, 12, 13, 14, 15, and 17 (Fig. 3C). Specifically, *PtrHAG7* and *PtrHAG2* were distributed on chromosome 2, while *PtrHAG1* and *PtrHAG6* were distributed on chromosome 5. *PtrHAC2* and *PtrHAF2* were distributed on chromosome 7, while chromosomes 4, 12, 13, 14, 15 and 17 contained only one *PtrHAT*. The distribution of these genes across the chromosomes was not uniform. The extensive distribution of *PtrHDACs* and *PtrHATs* in the genetic ancestor suggests that they may play important roles in the complex life process.

The expansion of plant gene families is primarily driven by segmental and tandem duplications. Gene sequences derived these expansions at the same locus often exhibit a high similarity [21]. To identify instances of duplication and expansion of *PtrHDACs* and *PtrHATs*, a collinearity analysis was conducted. The results demonstrated that both *PtrHDACs* and *PtrHATs* exhibited collinearity, with the majority of the linear genes located on different chromosomes (Fig. 3). An intra-species collinearity analysis revealed that there were six fragment repeat events in the *PtrHDACs* of *PtrHDT901, PtrHDT902, PtrHDT903, PtrHDA904, PtrHDA901, PtrHDA908, PtrHDA909, PtrHDA912*. Among these, *PtrHDT901, PtrHDT902* and *PtrHDT903* exhibited a linear correlation with each other. The *PtrHATs* exhibited four fragment repeat events, namely *PtrHAC1*/*PtrHAC2*, *PtrHAF1*/*PtrHAF2*, *PtrHAG1*/*PtrHAG2*, *PtrHAG6*/*PtrHAG7*. The *PtrHATs* on chromosome 2 were found to be closely related to the *PtrHATs* on chromosome 5. A comparative analysis of *HDACs* and *HATs* in other plants revealed that *PtrHDACs* and *PtrHATs* exhibited a strong collinearity with the majority of *HDACs* and *HATs* in *A. thaliana*, *Salix purpurea* and *M. domestica*, but little collinearity in *O. sativa.* In particular, more than 68% of *PtrHDACs* and *PtrHATs* genes exhibited a correlation with *S. purpurea* or *M. domestica*. This indicated a high degree of genetic conservation among woody plants. In comparison to *A. thaliana* and *O. sativa*, *P. trichocarpa* has evolved a unique set of genes, including *PtrHDA901, PtrHDA903, PtrHDA904, PtrHDA905, PtrHDA906, PtrHDA912, PtrHAG1, PtrHAG2, PtrHAG5, PtrHAF1, PtrHAF2* and *PtrHAC3*.

### *Cis-*acting element analysis of *PtrHDAC* and *PtrHAT* promoters

**Fig. 4:**
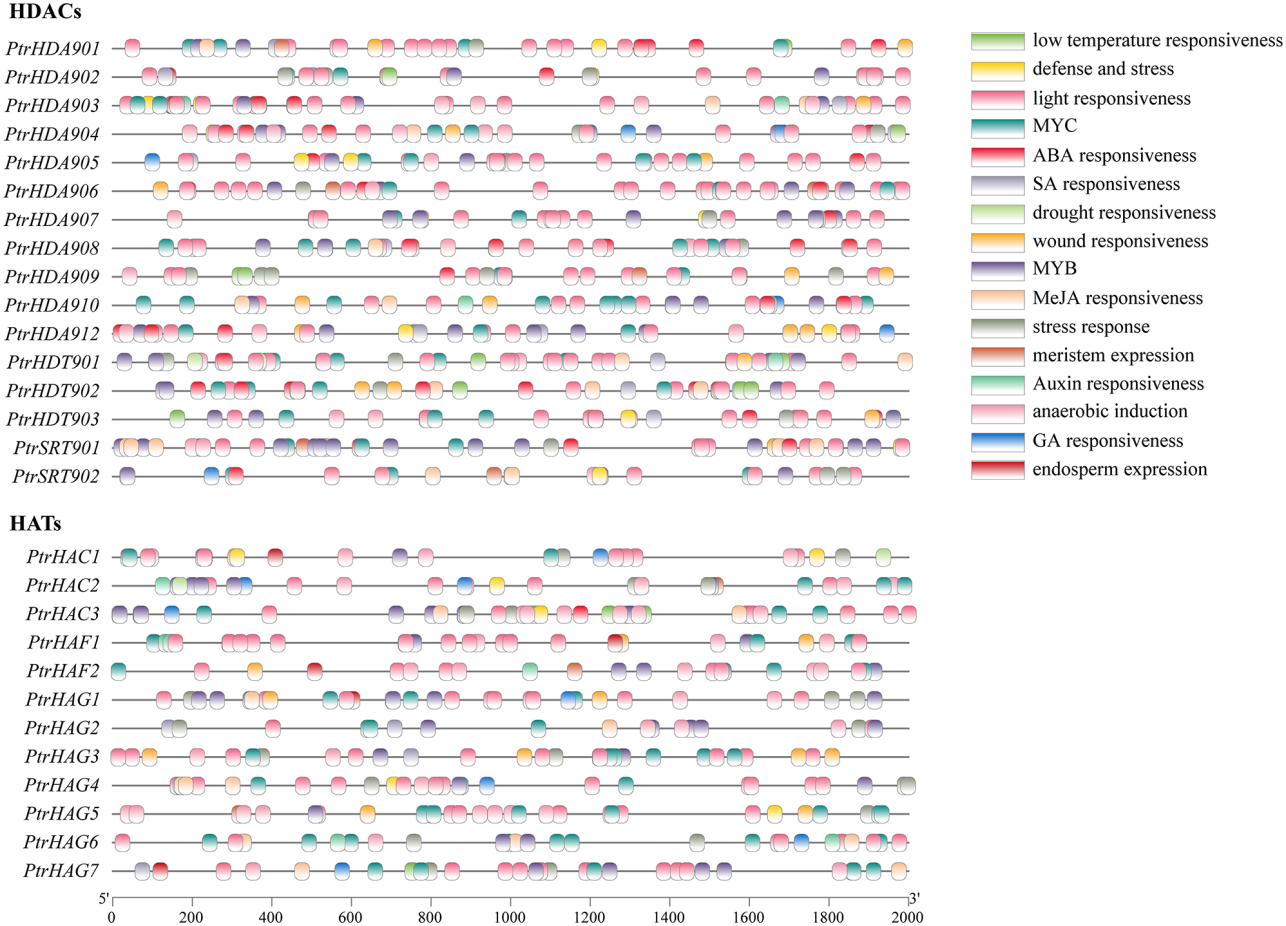
Cis-acting elements in *PtrHDACs* and *PtrHATs* promoter. Line represents 2000 bp gene upstream sequence, different color rectangles represent different response element

A 2000 bp sequence upstream of coding sequence (CDS) was extracted in order to predict *cis*-elements in *PtrHDACs* and *PtrHATs* (Fig. 4). In addition to the core *cis*-acting elements, the TATA-box and CAAT-box, the *PtrHDAC* and *PtrHAT* promoters also contained light response elements, stress response elements, hormone response elements, anaerobic induction response elements, seed-specific regulatory elements, and *cis*-regulatory elements. Among the *cis*-acting elements, the number of light-responsive elements was the highest, and each member of *PtrHDACs* or *PtrHATs* contained light-responsive elements. This suggests that PtrHDACs and PtrHATs possibly play important roles in the light-responsive pathway. Subsequently, 16 *PtrHDAC* family members were found to contain ABA response elements, 12 contained salicylic acid (SA) response elements, 9 contained methyl jasmonate (MeJA) response elements, 7 contained gibberellin response elements, and 5 contained auxin response elements. Each member of the *PtrHDAC* promoters contained an ABA response element. Except for *PtrHDA909*, the remaining members of the *PtrHDAC* family contained at least two *cis*-acting hormone elements. Seven *PtrHATs* promoters were found to contain ABA response elements, 7 genes contained SA and MeJA response elements, 7 genes contained gibberellin response elements and 4 genes contained auxin response elements. In the *PtrHAT* family, with the exception of *PtrHAG5* which lacked hormone response elements, the other family members were found to contain two or more hormone response elements. Previous studies have indicated that the expression of *PtrHDACs* and *PtrHATs* may be regulated by hormones.

Furthermore, the analysis of *cis*-elements revealed that the promoters of the *PtrHDACs* and *PtrHATs* contained a variety of stress response elements, such as drought response elements (MBS), low temperature response elements (LTR), wound response elements (WUN motif), and defense and stress response elements (TC-rich repeats). This indicated that the expression of *PtrHDACs* and *PtrHATs* was also induced by abiotic stress. This is consistent with the existing research indicating that *HDACs* and *HATs* play regulatory roles in a variety of abiotic stresses, either positively or negatively.

### Analysis of the HDAC and HAT interaction network in *P. trichocarpa*

**Fig. 5:**
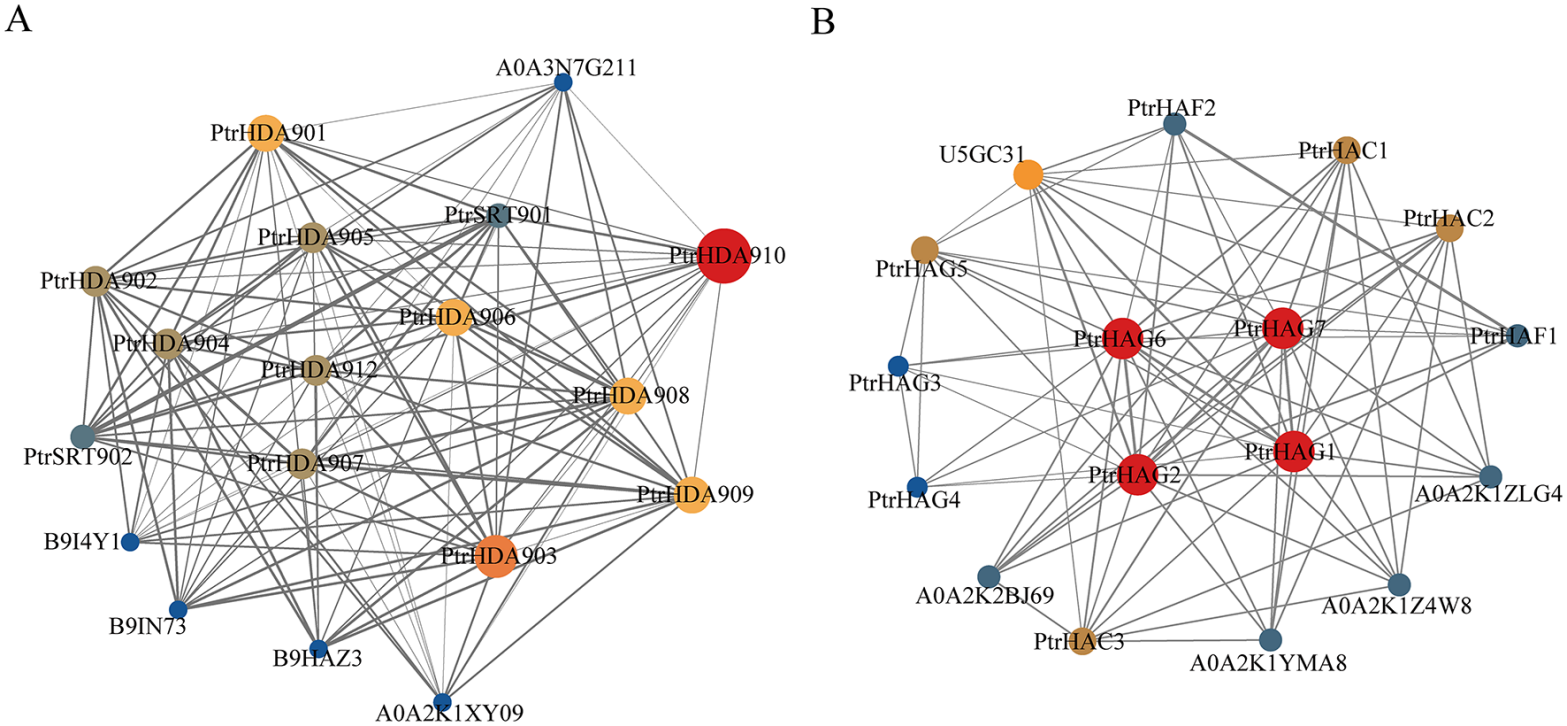
PtrHDAC (**A**) and PtrHAT (**B**) protein interaction network. The larger the node, the more the number of proteins that may interact. Thicker line segments indicate higher feasibility of interactions

The STRING database was employed to predict the interaction relationships of the PtrHDAC and PtrHAT family. A total of 18 nodes were identified within the PtrHDAC family, with a total of 116 groups of protein interactions observed among these nodes. A total of 17 nodes were identified in the protein interaction network of the PtrHAT family, with 80 groups of interaction relationships (Fig. 5). In the PtrHDAC family, except for the HD2 subfamily, there is a specific interaction between the other family members. The members of the RPD3/HDA1 subfamily engage in interactions with the members of the SIR2 subfamily, as well as with each other within the subfamily. Additionally, they may also interact with pathogenesis-related homeodomain protein (B9HAZ3 and B9IN73) and HhH-GPD domain-containing protein (B9I4Y1, A0A3N7G211 and A0A2K1XY09) (Fig. 5A). In the PtrHAT family, there was no interaction between CBP and TAFII250 members. However, it did interact with other subfamilies, and type I protein arginine methyltransferase (A0A2K1ZLG4, and A0A2K1YMA8), xyloglucan endotransglucosylase/hydrolase (A0A2K1Z4W8 and A0A2K2BJ69), and histone deacetylase domain-containing protein (U5GC31). Further details regarding the interaction network are provided in Table S3.

### Expression analysis of *PtrtHDACs* and *PtrHATs* in different tissues

**Fig. 6:**
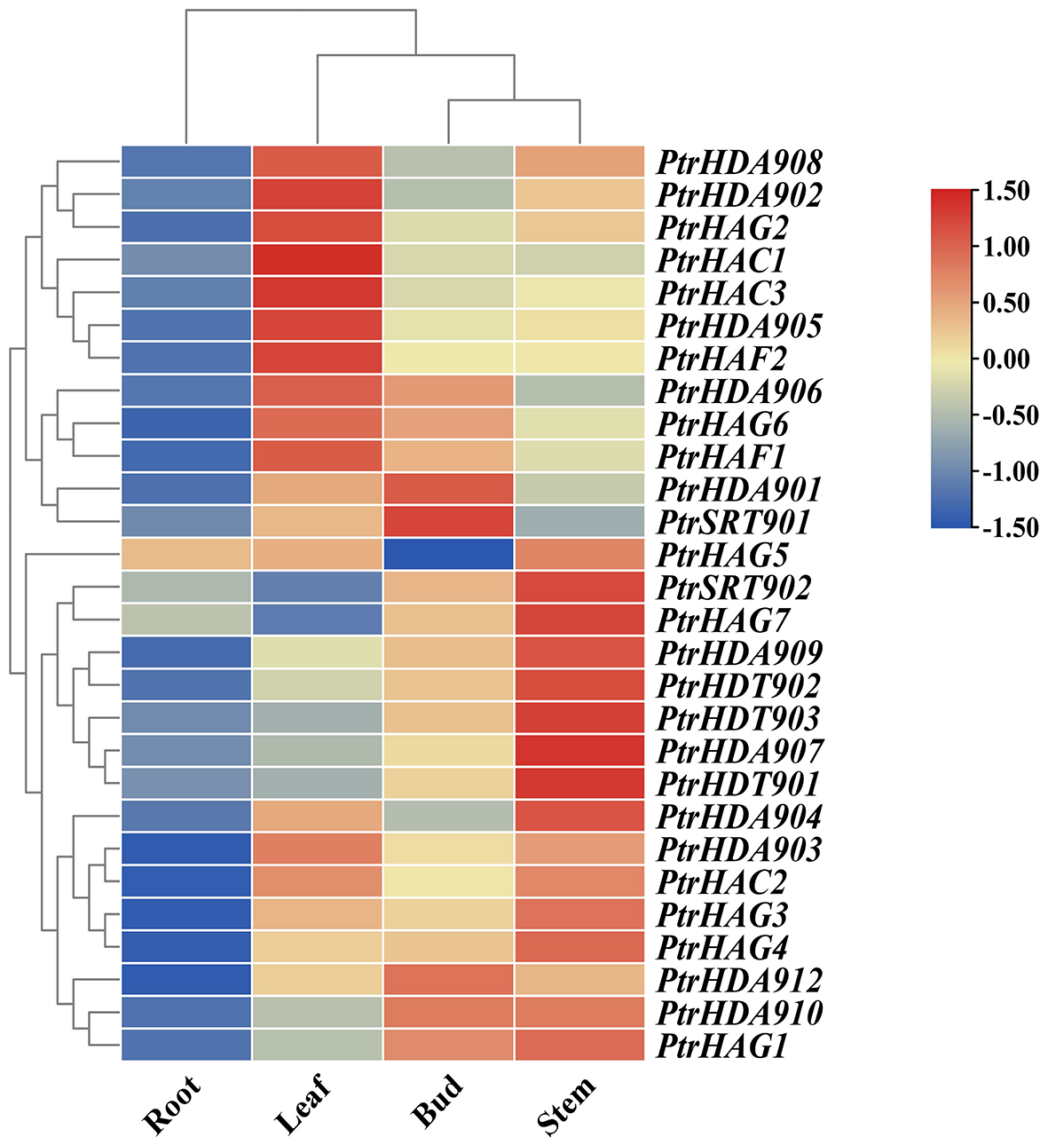
Heat map of the expression levels of *PtrHDACs* and *PtrHATs* in different tissues

In order to further insight into the functions of HDAC and HAT, the expression levels of *PtrHDACs* and *PtrHATs* were analyzed in roots, stems, leaves and apical buds (Fig. 6). The RPD3/HDA1 subfamily was predominantly expressed in leaves, with the highest expression observed for *PtrHDA906*. The HD2 subfamily members *PtrHDT901*, *PtrHDT902* and *PtrHDT903* exhibited the highest expression levels in stems, while the SIR2 subfamily members *PtrSRT901* and *PtrSRT902* exhibited the highest expression levels in apical buds. In the case of the HAT family, members of the CBP and MYST subfamilies were mainly expressed in leaves. The expression of *PtrHAG3* and *PtrHAG4* in the GNAT subfamily was observed to be higher in stems, while the expression of *PtrHAG6* was observed to be higher in leaves. The expression levels of the other members of the GNAT subfamily were found to be relatively low. However, the expression of *PtrHAF1* in the TAFII250 subfamily was high in leaves, while *PtrHAF2* was low in roots, stems, leaves and apical buds. The extensive expression of *HDAC* and *HAT* in different tissues indicated that *PtrHDACs* and *PtrHATs* may have distinct roles in the growth and development of poplar. The expression levels of all *PtrHDACs* and *PtrHATs* in roots were found to be extremely low, indicating that the acetylation modification occurred mainly in aerial tissues. This finding is consistent with the light response elements widely present in their promoters.

### Expression analysis of *PtrHDACs* and *PtrHATs* in response to drought stress

**Fig. 7:**
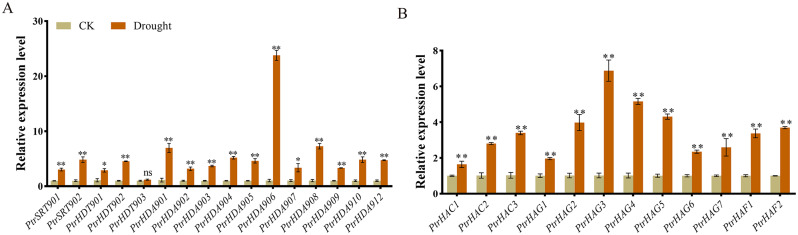
Expression analysis of *PtrHDACs* (**A**) and *PtrHATs* (**B**) in response to drought stress. Bars indicate means ± SE, *and ** show statistically significant differences (*p* < 0.05, Student’s *t* test, *n* = 3)

The identification of putative *cis*-regulatory elements in the promoter regions indicated that *PtrHDACs* and *PtrHATs* were involved in the response to abiotic stress. In this study, we took drought stress as an example (Fig. 7). The result demonstrated that the expression of *PtrHACs* and *PtrHATs* could be significantly induced by drought, with the exception of *PtrHDT903*. This suggests that acetylation modification plays an important role in the regulation of drought stress. In the HDAC family, the expression of *PtrHDA906* was significantly elevated under drought conditions, exhibiting a 24-fold higher than normal conditions (Fig. 7A). The expressions of *PtrSRT902*, *PtrHDA901, PtrHDA904, PtrHDA905, PtrHDA908, PtrHDA910*, and *PtrHDA912* were found to be upregulated by more than 5-fold. In the HAT family, the expression level of *PtrHAG3* was found to be significantly increased under drought conditions, with a 7-fold increase compared to normal conditions (Fig. 7B). The expression levels of *PtrHAG2, PtrHAG4, PtrHAG5*, and *PtrHAF2* were found to be upregulated by more than 4-fold. The results demonstrated that most of the *PtrHDACs* and *PtrHATs* were significantly upregulated in response to drought, indicating that the dynamics of histone acetylation and deacetylation, which are mediated by the two gene families, are important for poplar to cope with drought.

### Expression analysis of *PtrHDACs* and *PtrHATs* in response to hormone treatment under drought stress

**Fig. 8:**
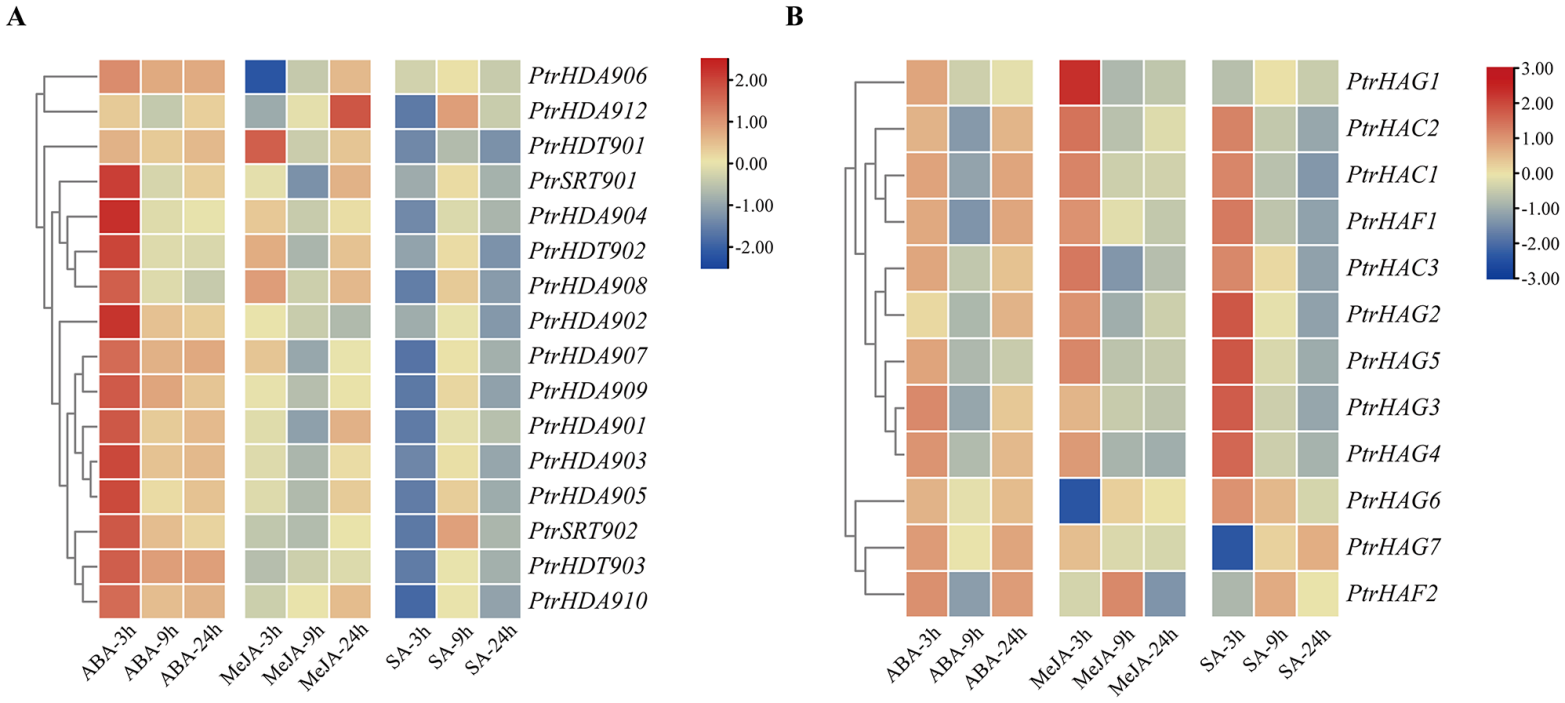
Heat map of the expression levels of *PtrHDACs* (**A**) and *PtrHATs* (**B**) at 3, 9, 24 h after hormone treatment (ABA, MeJA, SA) under drought conditions

The results demonstrated that the expression patterns of the *PtrHDAC* and *PtrHAT* families were distinct in response to different hormone treatments under drought stress (Fig. 8). Specifically, the ABA treatment resulted in a significant up-regulation of all *PtrHDACs*, with the exception of *PtrHDT901*, compared to drought conditions after 3 h. The expression level of *PtrHDA906* exhibited the most pronounced increase, up to 314-fold, suggesting a potential critical role of the gene in the ABA response to drought. The expression of *PtrHDT903*, *PtrHDA906*, *PtrHDA909*, and *PtrHDA910* was significantly induced following 9–24 h of ABA treatment. All *PtrHDACs* demonstrated a pattern of upregulation followed by downregulation in response to ABA treatment (Fig. 8A). In the HAT family, following 3 h of ABA treatment, *PtrHAG1* showed no significant change. In contrast, *PtrHAG7* and *PtrHAF2* exhibited a significant down-regulation, while the remaining *PtrHATs* exhibited a significant up-regulation. Among the genes whose expression was significantly altered following ABA spraying under drought stress, *PtrHAC3* exhibited the greatest upregulation, with a 51-fold increase in expression. Furthermore, the expression levels of *PtrHAC1, PtrHAC3, PtrHAG2, PtrHAG6* and *PtrHAF1* were found to be significantly increased following ABA treatment for 9–24 h. With the exception of *PtrHAG1, PtrHAG7* and *PtrHAF2*, the remaining *PtrHATs* showed up-regulation in response to ABA treatment during drought (Fig. 8B).

The expression levels of *PtrHDT901*, *PtrHDT902*, *PtrHDA906*, and *PtrHDA908* were observed to be elevated following a 3 h of MeJA treatment, in comparison to the sole drought treatment. *PtrHDT901* exhibited the most pronounced upregulation, reaching a 9-fold increase. The expression of *PtrHDA906* was the only one to be upregulated after 9 h, with a 44-fold increase in comparison to the only drought treatment. Furthermore, the expression of *PtrHDA906*, *PtrHDA910*, and *PtrHDA912* was found to be significantly increased following 24 h of MeJA treatment, with *PtrHDA906* exhibiting the highest upregulation, reaching 155-fold higher levels compared to the drought treatment. The expression patterns of *PtrHDACs* were found to be distinct in response to MeJA under drought conditions (Fig. 8A). *PtrHDT901* was induced at the early stage (3 h) of MeJA treatment, while *PtrHDA910* and *PtrHDA912* were induced at the later stage (24 h) of treatment, and *PtrHDA906* was continuously induced. In the HAT family, *PtrHAC1, PtrHAC2, PtrHAC3, PtrHAG1, PtrHAG2, PtrHAG5* and *PtrHAF1* exhibited significantly elevated expression levels following 3 h of MeJA treatment. It was observed that *PtrHAC3* exhibited the greatest induction, with a 76-fold increase relative to the sole drought treatment. *PtrHAC1, PtrHAC3, PtrHAG2, PtrHAG6*, and *PtrHAF1* demonstrated a significant up-regulation following 9–24 h of MeJA treatment. The expression of *PtrHAC1, PtrHAC2, PtrHAG1, PtrHAG5, PtrHAG6*, and *PtrHAF1* exhibited a biphasic pattern, first increasing and subsequently decreasing. Furthermore, *PtrHAC3* and *PtrHAG2* showed up-regulation followed by down-regulation and then up-regulation, while *PtrHAG3, PtrHAG4, PtrHAG7* and *PtrHAF2* were down-regulated.

In the SA treatment, only *PtrHDA906* was up-regulated after 3 h, with a 53-fold increase in expression compared to the only drought treatment. The expression of *PtrSRT902*, *PtrHDA906*, and *PtrHDA912* was increased after 9 h by SA treatment, with *PtrHDA906* exhibiting the greatest increase, 86-fold higher than that observed in drought conditions. All *PtrHDACs*, with the exception of *PtrHDA906*, exhibited a biphasic expression pattern in response to SA, initially displaying downregulation and subsequently upregulation (Fig. 8A). In the HAT family, with the exception of *PtrHAG1, PtrHAG7*, and *PtrHAF2*, the remaining genes exhibited increase after 3 h. Of these, *PtrHAC3* exhibited the greatest increase, reaching 66-fold higher levels than those observed under drought conditions. The expressions of *PtrHAC1, PtrHAC3, PtrHAG2, PtrHAG6*, and *PtrHAF1* were found to be significantly elevated for at least 9 and 24 h following the application of SA. All *PtrHATs* exhibited a biphasic expression pattern, initially up-regulated and subsequently down-regulated, with the exception of *PtrHAG1, PtrHAG7* and *PtrHAF2.*

## Discussion

Histone modification plays an important role in plant growth and development [22]. It has been demonstrated that histone transferase and deacetylase play roles in the plant physiological and metabolic processes [23]. In this study, a total of 16 *HDAC* and 12 *HAT* have been identified in *P. trichocarpa*. In the PtrHDAC family, the most member is the RPD3/HDA1 subfamily, while in the PtrHAT family, it is the GNAT subfamily. This pattern is similar to the found in *A. thaliana* [7], *Triticum aestivum* [24], and *Vitis vinifera* [25]. The results indicate that RPD3/HDA1 and GNAT may have undergone extensive expansions during the process of evolution [26]. The evolution of genomes and genetic systems is influenced by both tandem and segmental duplications, which contribute to the creation of new gene subfamilies [21]. The PtrHDAC family exhibited six segmental duplications. The PtrHAT family exhibited four segmental duplications. The gene pairs exhibited considerable similarities in both structure and function. However, it is possible that they may exhibit functional divergences in response to specific stimuli. For example, both *PtrHAF1* and *PtrHAF2* demonstrated notable alterations in response to drought. However, the expression of *PtrHAF1* increased in response to ABA, MeJA and SA compared to the drought, whereas *PtrHAF2* exhibited a decline in response to these hormones.

Both PtrHDACs and PtrHATs contain subfamily-specific structural domains. In addition, the subfamily members exhibit similar protein sequence lengths, motif compositions, and gene structures, suggesting a close phylogenetic relationship. In this study, the PtrHDACs were classified into three subfamilies: RPD3/HDA1, HD2, and SIR2, which is consistent with previous studies [27–31]. The data indicated that each subfamily contained conserved motifs and domains, which suggested that HDAC had undergone relatively little evolutionary change. The predicted protein interaction network indicates extensive interactions among the members of the PtrHDACs family, suggesting that HDACs may function collectively as complexes. For example, SlHDA1, SlHDA3, and SlERF.F12 form a ternary complex, which inhibited the transcription of maturation-related genes by reducing the level of histone acetylation in the promoter region [32]. The SiHDA9-SiHAT3.1-SiHDA19 trimer complex was found to be involved in the plant stress response through the deacetylation of H3K9 [33]. The PtrHATs were classified into four subfamilies: GNAT, MYST, CBP, and TAFII250. The results are consistent with the identified HATs in the genome of *A. thaliana* [7], *O. sativa* [34] and *M. domestica* [28]. Following an analysis of the protein structures of the largest subfamily of PtrHATs, it was observed that the structures of GNAT were markedly distinct, suggesting potential differences in function. Proteins on the same branch exhibit similar motifs and structures, suggesting that they perform analogous functions. In the predicted protein interaction network, in addition to the interaction between family members, PtrHATs may also interact with XTH proteins, suggesting that HAT could regulate plant growth and response to stress by participating in cell wall remodeling. Furthermore, 83% of PtrHATs may interact with U5GC31, which contains a histone deacetylase domain. This suggests that some HAT and HDAC may act in a synergistic manner.

Changes in gene structure can lead to differences in gene expression levels. In this study, the number of exons of *PtrHDACs* was 3 to 17, while the number of exons of *PtrHAT* was 9 to 21. The substantial disparity in the number of exons suggests that these genes exhibit disparate expression levels in distinct tissues. Some genes, such as P*trHDA902*, *PtrHAG2*, *PtrHAC1*, *PtrHDA905*, and *PtrHDA906*, exhibited high expression levels in the leaf but comparatively low expression in the root, bud, and stem. The observed expression patterns indicate that these genes may play a role in sensing environmental changes and participating in plant growth and stress response. HDA19, a homologous protein of PtrHDA902, is involved in the regulation of plant photomorphogenesis, and is required for plant tolerance to oxidative stress [35–37]. On the other hand, *PtrHDA909*, *PtrHDT902*, *PtrHDT903*, and *PtrHDA907* exhibited considerable expression activity in the stem. These genes may be involved in regulating material transport.

Gene expression is significantly affected by *cis*-acting elements within the promoter region [38]. Previous studies have demonstrated that *HDAC* and *HAT* genes play a significant role in the response of plants to various stresses and phytohormone treatments in plants [39, 40]. A total of five hormone-related elements and four stress-responsive elements were identified in the promoters of *PtrHDACs* and *PtrHATs*. The expression of all the genes was found to be altered by drought stress, in a manner consistent with the findings in *O. sativa*, *Hibiscus cannabinus* and *Dendrobium officinale* [3, 40, 41]. This is also linked to the presence of a drought response element, such as *PtrHDA901, PtrHDA903, PtrHDA906, PtrHDA910, PtrHDA912, PtrHDT901, PtrSRT901, PtrHAC1*, and *PtrHAC2*. Among these genes, the expression levels of *PtrHDA906* and *PtrHAG3* were the most increased in response to drought stress. It is expected that these genes exert their influence by regulating protein acetylation levels and affecting gene expression, in a manner analogous to their homologous genes [42, 43].

Phytohormones regulate the growth and development of plants, as well as their response to environmental stresses [44–46]. The application of exogenous hormones under conditions of drought stress has been demonstrated to increase the content of endogenous hormones and enhance the activity of carbon metabolism enzymes, thereby improving drought resistance [47]. Histone modifications can co-regulate plant responses to stress through synergistic interactions with phytohormone signaling pathways [39]. It has been reported that the H3K9ac of the core transcription factor of ABA signal transduction was significantly enriched under drought stress [48]. MdHDA6 was found to be significantly induced by drought or ABA treatment. It was also observed that MdHDA6 inhibits the expression of ABI5 downstream genes through histone deacetylation, thereby reducing drought tolerance [49]. Conversely, HDA704, which was significantly induced by drought and ABA, positively regulated the drought response through deacetylation of ABI5 [17]. Recruited by MYB96, AtHDA15 bound to the ABA negatively regulated genes *ROPs* and repressed their expression by deacetylation of histone H3 and H4. Those results are in the positive regulation of ABA response and plant drought tolerance [19]. *PtrHDA907*, the closest homolog homologue *AtHDA15*, was found to be upregulated by ABA under drought stress, suggesting the existence of analogous regulatory pathways. AtHD2C has the potential to enhance drought tolerance by inhibiting the expression of ABA-responsive genes (*ABI2*, *ADH1*, *KAT1* and *KAT*) [50]. However, ABA repressed the expression of *AtHD2C*, *HDT701* and *HDT702*, which similar to *PtrHDT901* was not induced by ABA [3]. HDA909 promoted the expression of ABA biosynthesis genes and the accumulation of ABA to improve drought tolerance in plants, which is consistent with the fact that *PtrHDA909* can be induced by ABA [20]. ABO1 encodes a homolog of the yeast histone HAT complex, which plays a biological role is through the acetylation of histones H3 and H4 in chromatin. The *abo1* mutant displayed a reduction in the expression of ABA-responsive genes that exhibited drought tolerance [51]. The expressions of *PtrHAC3*, *PtrHAG6* and *PtrHAF1* and *PtrHDACs* were induced by ABA under drought stress, indicating that they may respond to drought stress through the ABA signaling pathway. Combined with the existing reports, our findings indicate that ABA promotes the expression of PtrHDACs and PtrHATs in a relatively short period of time, and enhances drought resistance by modifying the acetylation level in plants, exemplified by *PtrHDA906*, *PtrHAC1*, *PtrHAC3*, *PtrHAG2, PtrHAG6*, and *PtrHAF1*.

In response to biotic or abiotic stresses, plants activate defensive responses through the production of MeJA, thereby increasing plant resistance. Exogenous spraying of MeJA promoted stomatal closure and improved drought resistance [52]. A number of studies have demonstrated that AtHDA6 played a role in the JA signaling pathway [18, 53–55]. HDA6 dissociated from the *PDC1*, resulting in an increase in histone H4 acetylation level. This activation of the JA signaling pathway subsequently improved the plant drought tolerance [18]. In addition, HDA6 and HDA19 were induced by MeJA and positively regulate the expression of JA-responsive genes, but their function in regulating JA signaling was independent on their HDAC activity [56]. JAZ proteins recruit HDA6 and deacetylate histones, thereby preventing ethylene-stabilized transcription factors (EIN3/EIL1) from binding to chromatin and facilitating the JA signaling pathway. Meanwhile, the level of H4 acetylation in the ERF1 promoter was diminished in the *ein3eil1*, indicating that histone acetyltransferases are implicated in the EIN3/EIL1-mediated gene activation process [54]. These results indicate that HDACs do not directly influence the acetylation levels of JA-responsive genes and are indirectly involved in the JA signaling pathway. This may be the reason why exogenous MeJA induced the majority of *PtrHATs*, while only a few *PtrHDACs* were induced. In particular, *PtrHAC1*, *PtrHAC3*, and *PtrHAF1* were continuously induced by MeJA in response to drought.

Exogenous application of SA can also alleviate plant drought stress [57]. The expression of SA biosynthesis genes and SA levels were found to be increased in the *hda19* mutant. It was also observed that these levels were co-mediated with HATs, which resulted in the expression of stress-responsive genes being increased levels of acetylation to improve plant resistance [58]. Similarly, HDA6 indirectly inhibited SA biosynthesis to maintain the low levels of SA required for normal plant growth by repressing the expression of the transcription factors CBP60g and SARD1 through deacetylation [59]. AtSRT2 was found to negatively regulate the expression of genes involved in the biosynthesis of SA, but its expression was not induced by exogenous SA [60]. Similarly, with the exception of *PtrHDA906*, the remaining *PtrHDACs* were not induced by SA. Furthermore, GCN5 has also been described as a repressor of the SA-mediated stress response [61]. Furthermore, some HATs are regarded as positive regulators in the SA signal pathway. HAC1 and HAC5 form a complex with other proteins to activate the transcription of stress response genes by reprogramming the histone acetylation [62]. On the other hand, SA induces the production of endogenous NO to influence histone acetylation, leading to the inhibition of HDACs and the hyperacetylation of histones [63]. The results of this study indicate that the majority of *PtrHDACs* were reduced expression levels during the SA treatment under drought stress, whereas the *PtrHATs* were the opposite pattern. These findings indicate that exogenous SA may enhance drought resilience in plants by elevating the acetylation level at the early stage of application.

## Conclusion

A total of 16 *PtrHDACs* were identified, consisting of 11, 2 and 3 members of the RPD3/HDA1, SIR2 and HD2 subfamilies, respectively. The 12 *PtrHATs* were comprised of 3, 2, 5 and 2 members of the CBP, MSYT, GCN5 and TAFII250 subfamilies, respectively. The members of the same subfamily exhibited conserved domains and motifs. *PtrHDACs* and *PtrHATs* were unevenly distributed on 8 and 9 chromosomes, respectively. Furthermore, the promoter regions of all genes were found to contain *cis*-acting elements for drought or hormone response. All *PtrHDACs* and *PtrHATs* were widely expressed in aboveground tissues with different expression profiles, indicating that these genes might be involved in the regulation of various aspects on poplar growth and development. The expressions of *PtrHDA906* and *PtrHAG3* were significantly induced by drought. Furthermore, the expression patterns after spraying different hormones under drought stress showed that *PtrHDA906, PtrHAC1, PtrHAC3, PtrHAG2, PtrHAG6* and *PtrHAF1* could be crucial regulators of the hormonal modulation on the drought response in plants. In summary, these results extend our comprehension of the epigenetic regulation by hormone in poplar.

## Methods

### Identification and classification of *PtrHDAC* and *PtrHAT* genes

The identification of *P. trichocarpa* HDAC and HAT was conducted in accordance with previously description [7, 34]. The HDAC and HAT protein sequences of *A. thaliana*, *M. domestica* and *O. sativa* were downloaded from Phytozome (https://phytozome-next.jgi.doe.gov/) and used as queries for searches against the *P. trichocarpa* genomes using BLASTP in NCBI (https://www.ncbi.nlm.nih.gov/). Furthermore, the HMM files (HDAC: PF00850, PF02146; HAT: PF00583, PF01853, PF08214, PF00240 and PF00439) of the conserved domain were downloaded from the Pfam database (http://pfam-legacy.xfam.org/). A Hidden Markov Model profile of the HMMER 3.0 software was employed to search for putative HDAC and HAT of *P. trichocarpa*, with an E-value threshold of 1e^− 5^. The results obtained by the two methods were integrated with those of previous studies to avoid repetition [64].

The fundamental physicochemical properties of PtrHDACs and PtrHATs were analyzed using ExPASy (https://www.expasy.org/), including the theoretical isoelectric point (pI), molecular weight and hydrophilicity [65]. The online software WOLF PSORT (https://www.genscript.com/wolf-psort.html) was employed to predict the subcellular localization of PtrHDAC and PtrHAT.

### Protein sequence alignment and phylogenetic tree construction

To investigate the evolutionary relationship, a multiple protein sequence alignment was performed for HAT and HDAC in *P. trichocarpa*, *M. domestica, O. sativa*, and *A. thaliana* using ClustalW with default parameters. An unrooted phylogenetic tree was constructed based on the neighbor-joining method with 500 bootstrap replicates using MEGA 11.0 [66]. The calculated results were visualized and optimized further optimized using the online tool Evolview.

### Phylogenetic tree analysis, motif and sequence structure analysis of PtrHDACs and PtrHATs

To analyze the homology of PtrHDACs and PtrHATs, the phylogenetic tree was constructed using the MEGA11.0. The protein sequence files of PtrHDACs and PtrHATs were uploaded by the MEME toolkit (https://meme-suite.org/meme/tools/meme), with the number of motifs to be searched was set to 20, and the remaining parameters were default [67]. The gene position information was downloaded. The gene names, motif analysis results, and gene structure annotation information for *PtrHDACs* and *PtrHATs* were imported simultaneously into the Gene Structure View tool of TBtools (v2.003) for visualization.

### Collinearity analysis of *PtrHDACs* and *PtrHATs*

Tandem and segmental duplications are major factors in the generation and maintenance of gene families, and genes on the same chromosome are considered to be tandemly duplicated genes [68]. Analysis of gene duplication events in *P. trichocarpa* HDAC and HAT family members using the multicollinearity toolkit MCScanX [69]. Collinearity analysis was performed between *P. trichocarpa* HDAC or HAT family members and other species including *A. thaliana*, *O. sativa*, *M. domestica* and *S. purpurea*. Collinearity plots were generated using the Circos tool and the multiple synteny plot tool in TBtools (v2.003) [70].

### Promoter *cis-*element analysis

The 2000 bp upstream sequences of the transcription start site were downloaded by TBtools (v2.003), and then the PlantCARE database (https://bioinformatics.psb.ugent.be/webtools/plantcare/html/) was used to analyze *cis*-regulatory elements [71]. Visualization was performed using TBtools (v2.003).

### Protein interaction network analysis

The protein interaction network of PtrHDAC and PtrHAT in *P. trichocarpa* was predicted by STRING (https://cn.string-db.org/) database [72]. The sequence of all proteins was used as query, and *P. trichocarpa* was selected as the “Organism”. The initial configuration settings were set to their default values, including the parameters “meaning of network edges,” “active interaction sources,” and “interaction score.” Subsequently, the protein interaction network was exported and visualized using Cytoscape (version 3.9.1).

### Plant materials and treatments

*Populus tomentosa* was propagated in woody plant medium (WPM) in a growth chamber (25℃ constant temperature, 16 h/8 h light-dark cycle, 10,000 lx light intensity, and 65% relative air humidity). Three-week-old tissue cultured plantlets were transplanted into soil (vermiculite: vegetative soil, 1: 2) in a greenhouse (25 °C, 16/8 h photoperiod). Following a period of one month of incubation, cuttings exhibiting uniform height and growth status were selected for the application of a drought treatment. Before the stress treatment the soil was fully watered to keep the moisture [73]. The cuttings were placed in an open and ventilated area for natural drying without watering. A control with normal watering was used. During drought experiment, water was replenished by weighing method at 10:00 every day to keep the relative moisture content of the soil within 40%, and the drought stress treatment was carried out for 4 days. According to the literature, we used three hormones (100 µM ABA, 500 µM SA, and 250 µM MeJA) sprayed until water droplets dripped from the leaf surface [74–76]. Hormone treatment was applied on the third day when the relative soil moisture content was 40%. After 0, 3, 9 and 24 h of hormone treatment, the leaves were sampled, frozen in liquid nitrogen and stored at -80 °C for total RNA extraction.

Roots, stems, leaves, and apical buds of three-week-old tissue cultured plantlets were collected and stored at -80 °C for analysis of *PtrHDACs* and *PtrHATs* expressions in different tissues after total RNA extraction.

### RNA isolation, reverse transcription, and RT-qPCR detection

Total RNA samples were extracted from leaves of *P. tomentosa* using the BIOFIT plant RNA extraction reagent kit (v1.6; Biofit Biotechnologies Co., Ltd., Chengdu, China). Total RNA was reverse transcribed into cDNA and digested the genomic DNA (gDNA) with the YEASEN Hifair^®^ III 1st Strand cDNA Synthesis SuperMix Kit (gDNA digester plus) (HB221101; Yeasen Biotechnology Co., Ltd., Shanghai, China) according to the manufacturer’s instructions.

The RT-qPCR experiments were performed using YEASEN Hieff^®^ qPCR SYBR^®^ Green Master Mix (HB210720; Yeasen Biotechnology Co., Ltd., Shanghai, China) with a BIO-RAD CFX96 Touch™ Real-Time PCR Detection System (Bio-Rad, USA). The RT-qPCR parameters were 95 °C for 2 min, followed by 95 °C for 10 s, 54 °C for 25 s and 72 °C for 30 s within 39 cycles. The relative gene expression was calculated according to the comparative CT method [77], and 18 S was used as an internal control. The RT-qPCR gene-specific primers are designed with Primer-BLAST in NCBI and listed in Table S1.

### Quantification and statistical analysis

All quantitative data were obtained from at least three biological replicates. The significant differential expression of genotypes under well-watered and drought-stress was obtained by one-way analysis of variance (ANOVA) in IBM SPSS26.0. Student’s *t* test was used for all pairwise comparisons, with *P* values < 0.05 considered as significant differences. Statistics were visualized using GraphPad Prism 8.0 software.

### Electronic supplementary material

Below is the link to the electronic supplementary material.

Supplementary Material 1

Supplementary Material 2

Supplementary Material 3

Supplementary Material 4

## Data Availability

The accession numbers for the genes used in our study are listed in supplementary Table 4. The datasets generated and analyzed during the current study are available from the corresponding author.

## Abbreviations

HDACs: Histone deacetylases
HATs: Histone acetyltransferases
RPD3/HDAC1: Reduced potassium dependency 3/histone deacetylase 1
HD2: Histone deacetylase 2
SIR2: Silent Information regulator 2
GCN5: General control non-depressible 5
GNAT: GCN5-related N-terminal acetyltransferase
CREB: cAMP-responsive element-binding protein
CBP: CREB-binding protein
MYST: MOZ, Ybf2/Sas3, Sas2, and Tip60
TAFII250: TATA-binding protein-associated factor
ABA: Abscisic acid
pI: Isoelectric point
CDS: Coding sequences
SA: Salicylic acid
MeJA: Methyl jasmonate
MBS: Drought response element
LTR: Low temperature response element
WUN: Wound response element
WPM: Woody plant medium
HMM: Hidden Markov Models
